# Tai Chi exercise improves age‐associated decline in cerebrovascular function: a cross‐sectional study

**DOI:** 10.1186/s12877-021-02196-9

**Published:** 2021-05-06

**Authors:** Lili Li, Jingjing Wang, Shaoying Guo, Yangqi Xing, Xiongwen Ke, Yinghao Chen, Yuan He, Shun Wang, Jiayu Wang, Xinwu Cui, Zhihua Wang, Lixu Tang

**Affiliations:** 1 Central Laboratory, Renmin Hospital of Wuhan University, 430060 Wuhan, China; 2 School of Physical Education, Wuhan Sports University, 430079 Wuhan, China; 3 Department of Cardiology, Renmin Hospital of Wuhan University, 430060 Wuhan, China; 4 Department of Medical Ultrasound, Tongji Hospital of Tongji Medical College of Huazhong University of Science and Technology, 430030 Wuhan, China

**Keywords:** Ageing, Cerebrovascular function, Healthy ageing, Tai Chi

## Abstract

**Background:**

Tai Chi exercise has been reported to enhance physical and mental health in the older adults; however, the mechanism remains elusive.

**Trial design:**

We recruited 289 older adults practicing Tai Chi for over 3 years, together with 277 age-matched older and 102 young adults as controls. 168 Tai Chi practitioners were successfully matched to 168 older controls aged 60–69 based on a propensity score for statistics.

**Methods:**

Cerebrovascular function was evaluated by measuring the hemodynamics of the carotid artery. Spearman correlation was performed to validate the age-associated physiological parameters.

**Results:**

Cerebrovascular function in older adults significantly degenerated compared with the young, and was substantially correlated with age. Compared with the older control group, Tai Chi practitioners showed significant improvements in CVHI (cerebral vascular hemodynamics indices) Score (*P* = 0.002), mean blood flow velocity (*P* = 0.014), maximal blood flow velocity (*P* = 0.04) and minimum blood flow velocity (*P* < 0.001), whereas the age-related increases in pulse wave velocity (*P* = 0.022), characteristic impedance (*P* = 0.021) and peripheral resistance (*P* = 0.044) were lowered.

**Conclusions:**

These data demonstrate a rejuvenation role of Tai Chi in improving the age-related decline of the cerebrovascular function.

**Trial registration:**

Chinese Clinical Trial Registry (ChiCTR1900025187)

## Background

A combination of remarkable increases in life expectancy and declining fertility rates results in a larger elderly population, a problem challenging economic development globally [1]. Although expected lifespan has continued to elongate in past decades, healthspan has barely increased [2]. Moreover, most efforts in research are dedicated to promote longevity, but few studies focus on improving healthy ageing [3, 4].

One major issue impeding the development of this field is the lack of reliable healthspan indicators or biomarkers. Age is an independent risk factor for cerebrovascular diseases, which dominate common health problems in the older adults [2]. Decline in cerebrovascular function is a leading cause for age-related cognitive deterioration, accompanying with high incidence of Parkinson disease, Alzheimer’s disease and stroke [5–8]. Age-related alterations in cerebrovascular function result in increased arterial stiffness, chronic vascular inflammation and endothelial dysfunction, and cause hypertension, arrhythmia, that commonly seen in the older adults [9, 10]. Hence, cerebrovascular functions reflect the health status in the older adults.

Regular physical activity is associated with improvement in physical health and protection against cerebrovascular diseases [11, 12]. Tai Chi is a famous exercise suitable for the older adults due to its slow and gentle movements [13]. A growing body of carefully conducted research has demonstrated the beneficial effects of Tai Chi on many conditions commonly associated with age, such as fall prevention, cognitive performance improvement, and muscle strength [14–18]. In addition to disease prevention, Tai Chi has been reported to be an effective treatment for certain neurodegenerative disorders like Parkinson disease and Alzheimer’s disease [15, 17, 19]. However, the mechanism whereby Tai Chi exerts beneficial effects on healthy ageing remains elusive.

Here we conducted a cross-sectional study to investigate the impact of Tai Chi practice on age-associated declines of cerebrovascular function by measuring the hemodynamics of the carotid blood flow. Our study provides direct evidence for the anti-ageing effect of Tai Chi through improving blood supply in the brain.

## Methods

### Trial design

From December 2017 to March 2018, we recruited older adult Tai Chi practitioners, together with age-matched older adults and young adults as controls in Wuhan, China to evaluate the effects of Tai Chi on age-related changes in cerebrovascular functions. This study was approved by Ethical Committees from both Renmin Hospital of Wuhan University and Wuhan Sports University. This study was registered on Chinese Clinical Trial Registry (http://www.chictr.org.cn; ChiCTR1900025187), and performed in accordance with the principle of the Helsinki Declaration II. Written informed consent was obtained from all participants in advance. Inclusion criteria were based on the following: healthy older people (60–69 years old), regular practice of Tai Chi more than three year for the Tai Chi group; healthy physical active older people (60–69 years old) for older adult control group; healthy young adults (21–25 years old) for young adult control group. Exclusion criteria included: cancer, surgery history, cognitive impairment, pregnant females, gastrointestinal diseases or had gastrointestinal surgery before, abnormal liver and kidney function, evidence of acute or chronic inflammatory or infectious diseases. This is a cross-sectional study. Data were collected in a double-blinded manner.

### Basic measurement

All the participants were given a clinical and physiological examination at the Physical Examination Centre, Renmin Hospital of Wuhan University (Wuhan, China). One day before testing, all participants were advised not to consume caffeine or alcohol, smoke or engage in vigorous exercise. Blood pressure in the arms was measured using an automated oscillometric device (OMRON-M6, Omron Healthcare, Vernon Hills, Illinois, USA). We recorded the sex, age, weight, systolic blood pressure (SBP), diastolic blood pressure (DBP), and heart rate and calculated body mass index (BMI).

### Measurement of cerebrovascular function

Left internal carotid artery hemodynamics were measured using a cerebrovascular function tester GT-3000 (Sino-medline, Beijing, China). The instrument has two probes: the Doppler probe and the pulsation pressure sensing probe. Both probes record parameters at the level of the internal carotid. Doppler probe records cerebral blood flow, cerebral blood flow rate and average blood flow. Pulse pressure sensing probe records cerebrovascular elasticity, external resistance, pulse wave velocity and other related indicators. The subjects were in a lying flat position. Cerebral vascular hemodynamics indexes (CVHI) were calculated. The recorded or calculated parameters include CVHI score, mean blood flow rate (Qmean), mean blood flow velocity (Vmean), maximum blood flow velocity (Vmax), minimum blood flow velocity (Vmin), pulse wave velocity (Wv), characteristic impedance (Zcv), dilatability (DI), resistance vascular (Rv), dynamic resistance (DR), critical pressure (CP) level, and diastolic pressure and critical pressure difference (DP).

### Statistical analysis

All data were processed using SPSS 22.0 (IBM Corporation, Armonk, New York, USA) and GraphPad Prism software 8.0 (San Diego, California, USA). Sample size was predicted by the following formular n=[(Z_α/2_+Z_β_) × σ/ δ]^2^×(Q1^− 1^+Q2^− 1^). To carry out stratified analyses, we performed the propensity score matching process while simultaneously forcing an exact match of sex, BMI, and age between older adult control group and the Tai Chi group. Then we compared matched groups within clinical parameters. All continuous variables were summarized as mean ± standard deviation (SD), and non-normally distributed continuous variables were summarized as median (25th - 75th ). The statistical difference in continuous variables normally distributed data between the two groups was compared using the student’s *t* test. The Mann-Whitney *U* test was used for comparisons between groups for non-normally distributed continuous variables. A correlation Spearman test was used to test the coefficients of non-normally distributed continuous variables. *P* < 0.05 was considered statistically significant. All statistical analyses were two-tailed.

## Results

### Validation of changes in cerebrovascular function during ageing

**Fig. 1:**
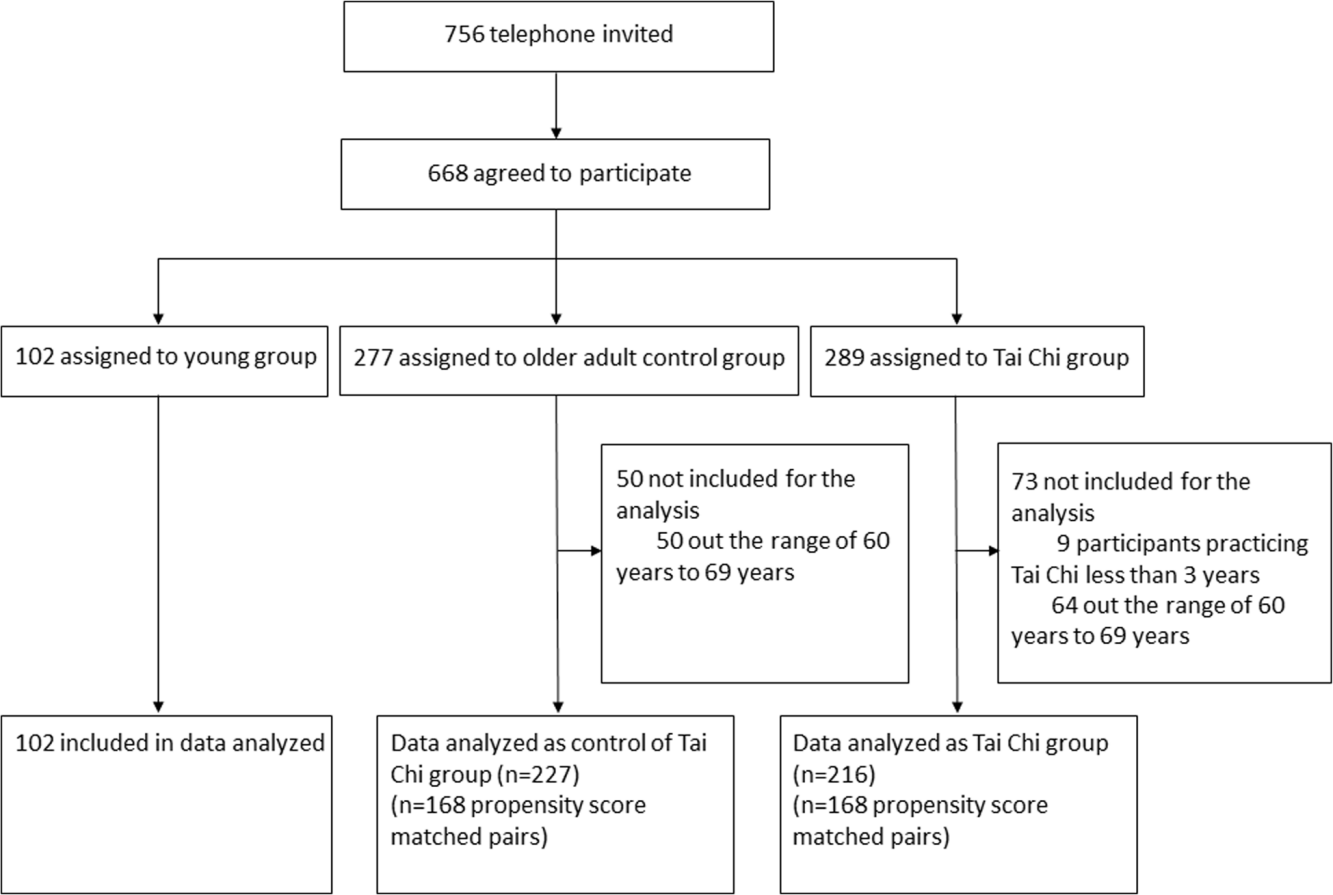
Flow chart of the study. Flow chart demonstrating the included subjects in each step of analysis during the entire procedure

We recruited a total of 668 participants, including 289 Tai Chi practitioners aged 49–76 (117 male/172 female), together with 277 people aged 51–73 (131 male/146 female) without any Tai Chi training before and 102 young adults aged 22–25 (49 male/53 female) (Fig. 1). Among the 168 age-matched participants practicing Tai Chi for over three year, 116 (69.05%) participants regularly practice Tai Chi every day, 38 (22.62 %) participants practice Tai Chi 3–5 times per week and 14 (8.33 %) participants practice Tai Chi twice per week. The exercise frequency and other physical activities being involved were listed in Table 1.

**Table 1:** Physical activities and exercise frequency of Young, Older and Tai Chi subjects

Group	Frequency	Tai Chi	Dancing	Jogging	Walking	Others
**Young (*n* = 102)**						
	2/week	2 (1.96 %)	1 (0.98 %)	10 (9.8 %)	0	12 (11.76 %)
	> 2/week	2 (1.96 %)	0	11 (10.78 %)	0	27 (26.47 %)
	everyday	6 (5.88 %)	0	9 (8.82 %)	0	17 (16.67 %)
	sum	10 (9.8 %)	1 (0.98 %)	30 (29.41 %)	0	56 (54.90 %)
**Older (*n* = 168)**						
	2/week	0	6 (3.57 %)	5 (2.98 %)	7 (4.17 %)	31 (18.45 %)
	> 2/week	0	10 (5.95 %)	5 (2.98 %)	5 (2.98 %)	14 (8.33 %)
	everyday	0	11 (6.55 %)	7 (4.17 %)	8 (4.76 %)	18 (10.71 %)
	sum	0	27 (16.07 %)	17 (10.12 %)	20 (11.91 %)	63 (37.5 %)
**Tai Chi (*n* = 168)**^**#**^						
	2/week	14 (8.33 %)	0	0	1 (0.60 %)	2 (1.19 %)
	> 2/week	38 (22.62 %)	3 (1.79 %)	0	0	1 (0.60 %)
	everyday	116 (69.05 %)	9 (5.36 %)	3 (1.79 %)	4 (2.38 %)	0
	sum	168 (100 %)	12 (7.14 %)	3 (1.79 %)	5 (2.98 %)	3 (1.79 %)

Data were shown as number (percentage). ^#^In the Tai Chi group, all of the participants regularly practice Tai Chi, whereas 36 subjects among them also participate in other sports practices

**Fig. 2:**
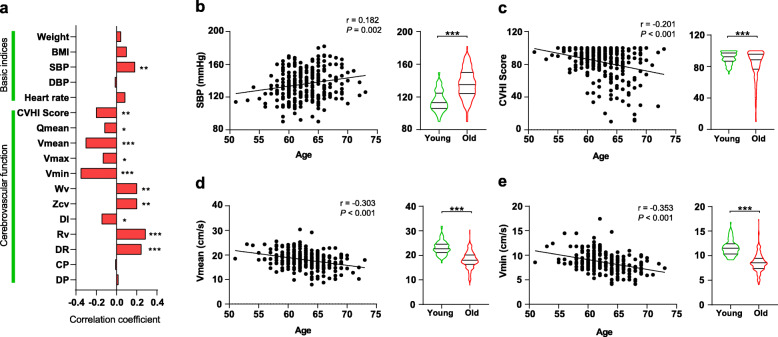
Cerebrovascular declines along with age . **a** Spearman’s correlation coefficient profiles of clinical indices with ages in older adults. ∗*P* < 0.05, ∗∗*P* < 0.01 and ∗∗∗*P* < 0.001. **b**-**e** Spearman’s correlation profiling (left) and comparisons between the young and the older adults (right) for age-related indices including SBP (**b**), CVHI Score (**c**), Vmean (**d**), Vmin (**e**). BMI, body mass index; SBP, systolic blood pressure; DBP, diastolic blood pressure; CVHI, cerebral vascular hemodynamics indices; Qmean, mean blood flow rate; Vmean, mean blood flow velocity; Vmax, maximum blood flow velocity; Vmin, minimum blood flow velocity; Wv, pulse wave velocity; Zcv, characteristic impedance; DI, dilatability; Rv, resistance vascular; DR, dynamic resistance; CP, critical pressure; DP, diastolic pressure and critical pressure difference. ∗∗∗*P* < 0.001

To validate which cerebrovascular parameters indeed reflect the health status of the older adults, we first examined the correlation between each parameter and age in older adult control group calculated by Spearman’s correlation coefficient (Fig. 2). As expected, SBP was significantly correlated with age in the older adults (correlation coefficient 0.182), and was significantly higher than young controls (*P* = 0.002; Fig. 2 a and b). Most of the cerebrovascular function parameters, including CVHI Score, Qmean, Vmean, Vmax, Vmin, Wv, Zcv, DI, Rv and DR, were significantly correlated with age in the older adults (Fig. 2 a), indicating a strong correlation between cerebrovascular function and age. Blood flow velocity Vmean and Vmin are indicators reflecting the blood flow status of cerebrovascular blood vessels. Lower speed of cervical arterial blood flow reflects the insufficient blood supply. We found that Vmean and Vmin ranked top with high correlation efficient [− 0.303 (*P* < 0.001) and − 0.353 (*P* < 0.001), respectively], and also significantly decreased in the older adults compared with young controls (Fig. 2 a and c-e). These data suggest that cerebrovascular function serves as a reliable marker to reflect the health status of the older adults.

### Tai Chi exercise improves the age‐related decline in cerebrovascular function

To evaluate the effects of Tai Chi exercise, we ran a matching program between Tai Chi group and the older adult control group aged 60–69 years old based on a propensity score to reduce the bias of age, sex and BMI. A total of 168 Tai Chi practitioners were successfully matched to 168 older adult controls, and were used for further analysis. There were no significant differences in basic physiological characteristics between the matched two groups (*P* > 0.05) (Table 2).

**Table 2:** Basal characteristics of the young and the matched older adult controls and Tai Chi players

	Young (*n* = 102)	Older (*n* = 168)	Tai Chi (*n* = 168)	*P* value
Age (y)	23.25 ± 1.11	63.7 ± 2.54	63.79 ± 2.51	0.762
Sex (female)	53 (52.0 %)	95 (56.5 %)	95 (56.5 %)	1
Body weight (kg)	62.96 ± 10.09	63.18 ± 10.35	63.92 ± 9.54	0.496
BMI (kg/m^2^)	22.18 ± 2.52	24.12 ± 3.09	24.17 ± 2.96	0.886
SBP (mmHg)	115.37 ± 12.74	137.55 ± 18.48	136.8 ± 16.38	0.691
DBP (mmHg)	68.98 ± 8.52	77.03 ± 10.62	78.08 ± 10.09	0.352
Heart rate (bmp)	69.35 ± 11.04	75.02 ± 11.65	73.51 ± 11.29	0.227

Values are mean ± SD or n (%)

*BMI* body mass index; *SBP* systolic blood pressure; *DBP* diastolic blood pressure

*P* values are calculated by comparing older adult control group with the Tai Chi group

CVHI Score is an overall evaluation score for cerebrovascular hemodynamic analysis. Compared with the older adult controls, Tai Chi practitioners showed a significant increase in CVHI Score [86.63 (72.25–95.00) in Tai Chi group and 91 (81.25–98.31) in older adults control group; *P* = 0.002], which almost approached the level in young control group [92.25 (86.63–97)] (Table 3). In detail, Tai Chi significantly increased the carotid blood flow velocity including Vmean (*P* = 0.014), Vmax (*P* = 0.04) and Vmin (*P* < 0.001). Wv and Zcv are indicators of the atherosclerosis degree of the brain, and associated with the overall elasticity of the arterial wall; and Rv reflects the degree of flow of small blood vessels and capillaries. We found that Tai Chi practice significantly reduced arterial resistance indices [Wv (*P* = 0.022), Zcv (*P* = 0.021) and Rv (*P* = 0.044)] (Table 3). These data suggest a rejuvenation of the carotid hemodynamics by Tai Chi exercise in the older adults.

**Table 3:** Impact of Tai Chi practice on cerebrovascular hemodynamics

	Young (*n* = 102)	Older (*n* = 168)	Tai Chi (*n* = 168)	*P* value
CVHI Score	92.25 (86.63–97)	86.63 (72.25–95)	91 (81.25–98.31)	0.002
Qmean (cm/s)	9.55 (8.42–11.2)	9.18 (8.48–10.31)	9.6 (8.73–10.39)	0.151
Vmean (cm/s)	22.72 (21.15–24.5)	17.58 (15.87–19.56)	18.35 (16.86–20.62)	0.014
Vmax (cm/s)	49.61 (45.91–53.51)	36.59 (33.23–39.91)	38.23 (33.92–41.43)	0.04
Vmin (cm/s)	11.52 (10.33–12.4)	8.27 (7.38–9.15)	8.56 (7.97–10.04)	< 0.001
Wv (m/s)	9.97 (7.99–12.44)	19.87 (15.5–23.96)	18.04 (14.19–22.24)	0.022
Zcv (kPa·s/m)	10.46 (8.4–13.06)	20.87 (16.28–25.16)	18.94 (14.9–23.36)	0.021
DI	0.96 (0.77–1.2)	0.53 (0.43–0.66)	0.54 (0.44–0.66)	0.606
Rv (kPa·s/m)	49.52 (44.18–56.78)	75.12 (64.4–90.8)	71.56 (63.69–81.15)	0.044
DR (kPa·s/m)	27.03 (23.71–31.71)	45.83 (34.88–54.69)	42.57 (34.34–52.45)	0.144
CP (kPa·s/m)	5.35 (4.14–6.27)	5.92 (4.51–7.05)	5.85 (4.46–6.91)	0.876
DP (kPa)	3.19 (2.72–3.82)	3.68 (2.94–4.49)	3.73 (3.05–4.48)	0.515

Data are median (25th -75th ). Variables are analyzed with Mann-Whitney U test for comparisons. *P* < 0.05 is considered statistically significant for two-tailed tests

*P* values are calculated by comparing older adults control group with the Tai Chi group. *CVHI* cerebral vascular hemodynamics indices; *Qmean* mean blood flow rate; *Vmean* mean blood flow velocity; *Vmax* maximum blood flow velocity; *Vmin* minimum blood flow velocity; *Wv* pulse wave velocity; *Zcv* characteristic impedance; *DI* dilatability; *Rv* resistance vascular; *DR* dynamic resistance; *CP* critical pressure; *DP* diastolic pressure and critical pressure difference

## Discussion

Ageing and age-related diseases have become the major threaten to human health [20]. Maintaining normal functional ability while getting old is the major goal to achieve healthy ageing. However, lack of clinical makers to indicate the health status in the older adults substantially prevents the development of this research field. Here we demonstrate that the cerebrovascular function represents a reliable indicator for healthy ageing, and Tai Chi exercise is effective to improve the age-related decline in cerebrovascular function.

Since cerebrovascular ageing is associated with cognitive decline, dementia and brain pathology, it is crucial to unveil the predictors of cerebrovascular function status for early detection and prevention [21]. Previous clinical evidence has shown that the cerebrovascular indices were highly sensitive to ageing [22, 23]. In the present study, we identified 11 cerebrovascular parameters that were significantly correlated with age in the older adults. CVHI score is a hemodynamic indicator reflecting the cerebrovascular structure and function, and is closely related to the occurrence of cerebrovascular diseases. Lower Vmax and Vmin in the aged people suggest that the blood supply is frequently insufficient, a possible reason for the high incidence of neuronal diseases in the older adults.

Arterial hemodynamics indices Vmean, Vmax, and Vmin are main parameters reflecting diastolic blood flow velocity. Cerebral dynamic Wv and Zcv are related to the elasticity of the arterial wall. Lowered Wv and Zcv indicate the better overall elasticity of the arteries. Rv is a quantitative indicator of the degree of the flow of small blood vessels and capillaries. Compared with the older adult controls, those who have practiced Tai Chi for 3 years showed alterations in most of the cerebrovascular parameters towards the young controls (Table 3). Firstly, these findings prove the effectiveness of Tai Chi to improve the cerebral health status in the older adults. Secondly, these findings support that the cerebrovascular parameters might serve as reliable indicators for healthy ageing. Thirdly, our study implicates that the age-related decline in cerebrovascular function is a reversible process. These ideas would prompt novel strategies to develop anti-ageing products.

Tai Chi has many positive effects on the health of the older adults, including improved motor ability, exercise efficiency and emotional state [24, 25]. In this study, we found that Tai Chi-style physical activity could prevent the ageing-induced cerebrovascular function decline of the older adults, which may effectively reduce the related cerebral diseases [16, 26–28]. In addition to the treatment of specific diseases, Tai Chi practitioners provide a cohort of healthy ageing population, which would be an irreplaceable opportunity to investigate the molecular mechanisms of ageing in human.

This study also has some limitations. Firstly, this is a cross-sectional study involving a cohort of Tai Chi practitioners with variable durations of Tai Chi practicing. The impact of duration or intensity of Tai Chi practicing on cerebrovascular function need to be further investigated. Secondly, we did not select another style of exercise as a strict control for Tai Chi. Thus, the observed changes would also reflect a general impact of regular physical activity. Thirdly, we did not discriminate the subtypes of Tai Chi they practiced, and could not calculate the session volume or duration during each practice. It would be impossible for them to insist on a constant manner to exercise during such a long period. Finally, the conclusions need to be further validated by a longitudinal cohort with well-controlled Tai Chi training.

Taken together, in this cross-sectional study, we found that the age-related decline in cerebrovascular hemodynamics in the older adults is significantly improved by Tai Chi practice. Our findings provide novel insights into healthy ageing and anti-ageing strategies.

## Data Availability

The datasets used and/or analysed during the current study are available from the corresponding author on reasonable request.

## Abbreviations

SBP: Systolic blood pressure
DBP: Diastolic blood pressure
BMI: Body mass index
CVHI: Cerebral vascular hemodynamics indexes
Qmean: Mean blood flow rate
Vmean: Mean blood flow velocity
Vmax: Maximum blood flow velocity
Vmin: Minimum blood flow velocity
Wv: Pulse wave velocity
Zcv: Characteristic impedance
DI: Dilatability
Rv: Resistance vascular
DR: Dynamic resistance
CP: Critical pressure
DP: Diastolic pressure
